# Secondary diabetes mellitus in pheochromocytomas and paragangliomas

**DOI:** 10.1007/s12020-023-03492-7

**Published:** 2023-09-20

**Authors:** Melpomeni Moustaki, Stavroula A. Paschou, Elena Vakali, Paraskevi Xekouki, Georgia Ntali, Evanthia Kassi, Melpomeni Peppa, Theodora Psaltopoulou, Marinella Tzanela, Andromachi Vryonidou

**Affiliations:** 1 Department of Endocrinology and Diabetes Center, Hellenic Red Cross Hospital, Athens, Greece; 2 Endocrine Unit and Diabetes Center, Department of Clinical Therapeutics, Alexandra Hospital, School of Medicine, National and Kapodistrian University of Athens, Athens, Greece; 3 Department of Endocrinology and Diabetes, University General Hospital of Heraklion, School of Medicine, University of Crete, Heraklion, Greece; 4 Department of Endocrinology and Diabetes Center, Endo ERN Center, Evaggelismos Hospital, Athens, Greece; 5 Endocrine Unit, First Department of Propaedeutic and Internal Medicine, Laiko Hospital, School of Medicine, National and Kapodistrian University of Athens, Athens, Greece; 6 Endocrine Unit and Diabetes Center, Second Department of Internal Medicine, Attikon University Hospital, School of Medicine, National and Kapodistrian University of Athens, Athens, Greece

**Keywords:** PPGLs, Secondary diabetes mellitus, Adrenergic phenotype, Noradrenergic phenotype, Aerobic glycolysis, Impaired insulin secretion

## Abstract

Secondary diabetes mellitus (DM) in secretory pheochromocytomas and paragangliomas (PPGLs) is encountered in up to 50% of cases, with its presentation ranging from mild, insulin resistant forms to profound insulin deficiency states, such as diabetic ketoacidosis and hyperglycemic hyperosmolar state. PPGLs represent hypermetabolic states, in which adrenaline and noradrenaline induce insulin resistance in target tissues characterized by aerobic glycolysis, excessive lipolysis, altered adipokine expression, subclinical inflammation, as well as enhanced gluconeogenesis and glucogenolysis. These effects are mediated both directly, upon adrenergic receptor stimulation, and indirectly, via increased glucagon secretion. Impaired insulin secretion is the principal pathogenetic mechanism of secondary DM in this setting; yet, this is relevant for tumors with adrenergic phenotype, arising from direct inhibitory actions in beta pancreatic cells and incretin effect impairment. In contrast, insulin secretion might be enhanced in tumors with noradrenergic phenotype. This dimorphic effect might correspond to two distinct glycemic phenotypes, with predominant insulin resistance and insulin deficiency respectively. Secondary DM improves substantially post-surgery, with up to 80% remission rate. The fact that surgical treatment of PPGLs restores insulin sensitivity and secretion at greater extent compared to alpha and beta blockade, implies the existence of further, non-adrenergic mechanisms, possibly involving other hormonal co-secretion by these tumors. DM management in PPGLs is scarcely studied. The efficacy and safety of newer anti-diabetic medications, such as glucagon-like peptide 1 receptor agonists and sodium glucose cotransporter 2 inhibitors (SGLT2is), as well as potential disease-modifying roles of metformin and SGLT2is warrant further investigation in future studies.

## Introduction

Pheochromocytomas and paragangliomas (PPGLs) are tumors arising from adrenomedullary or extra-adrenal chromaffin tissue of sympathetic or parasympathetic ganglia that commonly oversecrete one or more catecholamines: adrenaline, noradrenaline, and dopamine [1, 2]. PPGLs are rare, with a reported annual incidence of 0.6 ×10^5^ [3], as well as highly heritable, with germline mutations detected in one third of cases [2]. Depending on their genetic background, 70% of PPGLs can be classified in 3 clusters, namely pseudohypoxia-related, kinase signaling-related and Wnt-related, with strong genotype-phenotype correlation in terms of secretory profile and biological behavior. Cluster 1 tumors are characterized by noradrenergic phenotype, cluster 2 by adrenergic phenotype, and cluster 1 and 3 exhibit higher malignant potential [2]. Adrenergic phenotype corresponds to elevation of both catecholamines, with a relative increment of >5% of adrenaline over noradrenaline. Noradrenergic phenotype refers to predominant elevation of noradrenaline with no or relatively small increase of adrenaline [2].

Normally, catecholamines mediate the fight or flight response, i.e. the physiological reaction to harmful event, attack, or threat to survival, upon acting on their receptors, alpha 1 (α_1_), alpha 2 (α_2_), beta 1 (β_1_), beta 2 (β_2_) and beta 3 (β_3_) adrenergic receptors (ARs), as well as on dopamine receptors type 1 and 2 [4–6]. The end purpose of the effects of catecholamines is to optimize muscle performance [6]. This is achieved by a coordinated increase in blood flow (via vasoconstriction, tachycardia), oxygen delivery (via bronchodilation), and energy supply (via increased level of blood glucose and free fatty acids, FFAs). Therefore, autonomous catecholamine excess in secretory PPGLs leads to clinical presentations of hypertension, tachycardia, and hyperglycemia. The latter occurs approximately in up to 49.5% of PPGLs at a varying degree of severity, ranging from impaired glucose tolerance (IGT) (19.1–34.78%) [7, 8] and diabetes mellitus (DM) (23.4–48%) ([7–11] to few reported cases of diabetic ketoacidosis (DKA) or hyperosmolar hyperglycemic state (HSS) [12–16].

Secretory PPGLs occasionally co-secrete multiple other hormones and peptides, like adrenocorticotropic hormone (ACTH) or rarely corticotropin releasing hormone (CRH), somatostatin (SS), vasoactive intestinal peptide (VIP), parathyroid hormone (PTH), parathyroid hormone-related peptide (PTH-rP), enkephalins, endorphins, insulin-like growth factor 2 (IGF-2), chromogranin A (CgA), calcitonin (CT), CT-related peptide, atrionatriuretic peptide (ANP), and neuropeptide Y (NPY); these molecules may also affect glucose metabolism [17–21]. Moreover, pheochromocytoma has been reported to stimulate autonomous cortisol secretion in paracrine manner [22]. Indeed, discussing the effects of the above molecules in glucose metabolism is beyond the scope of this review.

Despite the high prevalence of glucose metabolism disorders in PPGLs, the interaction between catecholamines and glucose metabolism is not entirely understood. According to a recent clinical study in non-diabetic patients, adrenaline is shown to mainly inhibit insulin secretion, while noradrenaline appears to principally impair insulin sensitivity in parallel with stimulating insulin secretion [23]. In this review, we aim to explore the impact of adrenaline and noradrenaline on glucose metabolism at molecular level, amalgamating the evidence acquired from preclinical and clinical studies, in order to assess the pathogenetic basis and clinical relevance of this dimorphism of effect. Furthermore, we will discuss the presentation, prognosis and management of secondary DM in this clinical setting.

## Methods

Authors collected, analyzed and qualitatively resynthesized information regarding secondary diabetes mellitus in pheochromocytomas and paragangliomas. The impact of adrenaline and noradrenaline on glucose metabolism at molecular level, as well as evidence from preclinical and clinical studies was explored. English language literature was searched in PubMed until June 2023 using combinations of relevant terms.

## Insulin sensitivity

Catecholamine excess is a hypermetabolic state, characterized by enhanced energy expenditure and insulin resistance [5]. The former is shown by resting energy expenditure (REE) and respiratory quotient (RQ) in direct calorimetry [24], while the latter is demonstrated by post-operative increase of insulin sensitivity indices, such as glucose infusion rate (GIR) required to maintain euglycemia and insulin sensitivity index (ISI) during euglycemic hyperinsulinemic clamp [21, 25].

Insulin resistance is mediated both directly, via AR stimulation of target tissues, and indirectly, via α_1_ and β_2_- AR stimulation of glucagon secretion from alpha pancreatic cells [26]. Adrenaline is demonstrated to decrease GIR, as well as to increase endogenous glucose production (EGP) and glucagon level, upon β-AR activation [27]. The central role of β-AR in catecholamine-induced insulin resistance is reflected at the fact that α-blockers are not effective in improving insulin sensitivity, unless combined with β-blockers [21, 28]. This concept is further supported by studies with various adrenergic agonists, in which the induced increase in glucose release (from glycogenolysis and gluconeogenesis) is directly related to the specific β_2_-AR activity of each compound [5]. However, despite the improvement in insulin sensitivity by alpha and beta blockade, it is less substantial compared to surgical removal of PPGL [21].

In a recent cross-sectional study of a general Japanese population, a significant correlation between urine normetanephrine level and homeostatic model assessment of insulin resistance (HOMA-IR) is found, suggesting that catecholamines might affect glucose homeostasis even at levels in the upper end of the normal range [29].

### Muscle

As per their teleological purpose, catecholamines adapt glucose metabolism in order to meet the increased energy requirements of skeletal muscle during ‘’fight or flight” response. Thus, glucose uptake is expected to be increased upon activation of α_1_-AR and β_2_-AR, the 2 principal types of ARs expressed in skeletal muscle. Both adrenaline and noradrenaline activate these receptors, with the former bearing a higher affinity for β_2_-AR and the latter for α_1_-AR [26].

Upon β-AR stimulation (by adrenaline, isoprenaline and BRL37344), glucose transporter 4 (GLUT-4) is translocated to the plasma membrane, as shown in vitro, ex vivo, and in vivo [30–32]. However, the net effect of β-AR stimulation in glucose uptake and tolerance among these studies is controversial. In the first study, adrenaline is shown to substantially decrease glucose uptake ex vivo, in parallel with increasing GLUT-4 translocation; this dichotomy is attributed to decreased intrinsic activity of GLUT-4 (intrinsic activity hypothesis) [30]. In contrast, the second study demonstrates that isoprenaline and BRL37344 increase glucose uptake (in vivo, ex vivo, and in vitro) and tolerance (in vivo). This effect is associated with GLUT-4 translocation and involves mammalian target of rapamycin complex 2 (mTORC2) activation and cyclic adenosine monophosphate (cAMP) signaling [32]; yet, the exact mechanism is not clear [31]. The discrepancy between the above mentioned studies may be due to different adrenergic agonists used: in response to different ligands, β_2_-AR may preferentially activate different signaling pathways [31]. The third study sheds light into a possible interaction between β-AR and insulin signaling. Specifically, adrenaline is shown to increase basal glucose transport (by 20%) in the absence of insulin and to decrease insulin-stimulated glucose transport (by 50%) in the presence of insulin [32]. In concordance with these findings, two additional studies indicate that in the presence of insulin (during euglycemic hyperinsulinaemic clamp), epinephrine substantially decreases glucose disposal (by 50–75%) [33, 34].

Regarding α_1_-AR effects, data from transgenic mice models with constitutively active mutant (CAM) and knockout (KO) forms of α_1A_-AR and α_1B_-AR, demonstrate that α_1_-AR stimulation leads to increased glucose uptake ex vivo, accompanied by increased glucose tolerance in vivo [35].

Considering that adrenaline-secreting PPGLs are frequently accompanied by impaired insulin secretion (see Insulin secretion section), glucose uptake would be increased due to α_1_-AR and β_2_-AR stimulation. In addition, increased glucose uptake due to α_1_-AR is expected in PPGLs with noradrenergic phenotype. However, given that both ARs desensitize after prolonged exposure to catecholamines [36, 37], the above effects might wean off over the course of the disease [38].

Patients with noradrenergic phenotype are shown to preferentially use carbohydrates as energy substrates [24]. In both secretory phenotypes, insulin-mediated glycogenesis is inhibited and glycogenolysis is induced, by inactivation of glycogen synthase and activation of phosphorylase respectively [5, 33]. In addition, glucose-6-phosphate is activated and metabolism is shifted from oxidative phosphorylation to aerobic glycolysis (Warburg effect), resulting into increased release of lactate and alanine in the bloodstream [5, 33, 34]. In support of this, hepatic catheterization studies in humans demonstrate that adrenaline increases lactate concentration in the peripheral venous blood at a greater extent than in the hepatic venous blood [39]. During catecholamine crisis, excessively increased lactate concentration may lead to lactic acidosis [40].

### Adipose tissue

In order to serve ‘’fight or flight” response, catecholamines are expected to increase lipolysis in adipose tissue in order to generate energy substrates in the form of FFAs, and to simultaneously inhibit glucose uptake, so that glucose is spared for skeletal muscle and vital organs. Patients with adrenergic phenotype are shown to preferentially use lipids as energy substrates [24]. Adipose tissue is avid in α_1_ -AR and β_3_-AR. Adrenaline and noradrenaline have the same affinity for β_3_-AR, while noradrenaline has higher affinity for α_1_ -AR.

In response to β-AR stimulation (isoproterenol), there is enhanced lipolysis and impaired glucose uptake, as shown in vitro [41–43]. Interestingly, these 2 events seem to be interrelated, with lipolysis being a pre-requisite for inhibition of insulin-stimulated glucose uptake, given that the latter is reversed in the absence of the former, i.e. in adipocytes not expressing adipose triglyceride lipase (ATGL) or treated with lipase inhibitor. The underlying mechanism employs the inhibition of phosphatidylinositol-3 kinase (PI3K) - protein kinase B (Akt) - mTOR pathway at the level of mTOR (mTORC1 and -2) through complex dissociation by lipolytic products. In turn, mTOR inhibition blocks insulin signaling downstream and ultimately GLUT-4 translocation, leading to impaired glucose uptake [41]. Two other in vitro studies in rat adipocytes conclude that the effect of β-AR stimulation on glucose uptake is biphasic and dose-dependent. At low doses of isoproterenol, basal glucose transport and GLUT-4 translocation increase (by 40–60% and 90% respectively), likely via cAMP signaling [42, 43]. In contrast, at high doses of isoproterenol, basal and insulin-stimulated glucose transport decrease (by 40–55%) [42, 43]; in parallel, cAMP-mediated translocation of GLUT-4 is abolished but plasma membrane GLUT-4 content remains stable. This points towards a defect in the intrinsic activity of GLUT-4 [43], similarly with observations in muscle cells [32]. It should be underlined that β_3_-AR is not desensitized, thus the above effects are sustained during chronic catecholamine exposure [44]. Putting the above knowledge into clinical context, the ‘’low dose scenario” resembles the small increase in catecholamine level induced by exercise and explains why selective β_3_-AR agonists were studied as potential anti-diabetic agents in the past [44]. On the other hand, secretory PPGLs fit more into the ‘’high dose scenario”, being characterized by impaired glucose tolerance and excessive lipolysis; the latter may predispose to DKA, especially if combined with impaired insulin secretion.

Similar to skeletal muscle, α_1_-AR stimulation is demonstrated to increase glucose uptake in adipose tissue ex vivo, in transgenic mice that express a CAM form of the receptor (α_1A_-AR and α_1B_-AR isoforms), as well as in vivo, in humans treated with phenylephrine [35, 45]. In addition, glucose utilization is shifted to glycolysis, resulting into increased release of lactate [45]. Furthermore, α_1_ -AR stimulates lipolysis, which, interestingly, occurs in parallel with increased leptin secretion, further amplifying the above lipolytic effect via adenosine monophosphate-activated protein kinase (AMPK) signaling [35]. Albeit, considering that α_1_-AR is desensitized after prolonged exposure to catecholamines [36], the above effects might wean off over the course of the disease.

Furthermore, catecholamines have been speculated to alter adiponectin expression, with inconsistent findings amongst studies. In two studies, patients with pheochromocytoma have lower adiponectin level in comparison to normal-weight hypertensive patients and age, sex and body mass index (BMI) – matched healthy controls [46, 47]. Of note, adiponectin level increases postoperatively [46, 47], in conjunction with HOMA-IR decrease, despite weight gain [46]. As per the underlying mechanism, β-AR stimulation by isoproterenol in 3T3-L1 adipocytes is shown to down-regulate adiponectin expression via a G_S_-protein-PKA-dependent pathway in dose-dependent manner, with significant effect starting at low doses [48]. In contrast, a third clinical study associating adiponectin level with secretory profile of pheochromocytomas reveals that patients with noradrenaline-secreting tumors have 3-fold higher adiponectin level than controls [49]. In addition, a fourth clinical study fails to demonstrate a significant change in adiponectin level before and after surgery for pheochromocytoma [50].

Moreover, resistin, an insulin-antagonizing adipokine, is shown to be higher in patients with pheochromocytoma and DM than in non-diabetic ones, with its level being decreased post-surgery [47].

Finally, secretory PPGLs lead to subclinical inflammation, as shown by increased c-reactive protein (CRP) level [50] and tumor necrosis factor A (TNF-α) [47], which is recognized to be associated with insulin resistance [51].

Finally, few PPGLs have been shown to bear active brown adipose tissue (BAT) in 18F-fluoro-2-deoxy-D-glucose positron emission tomography-computed tomography (18F-FDG PET/CT), which occurs due to β_3_-AR stimulation by noradrenaline or due to other ‘’browning factors” secreted from PPGLs or is related to succinate dehydrogenase (SDH) mutations. Interestingly, these patients have higher mortality rate in comparison to age, gender, and BMI-matched PPGLs controls; the explanation of this phenomenon is not clear and might involve increased sympathetic tone, increased host stress, cachexia and wasting [52]. To the best of our knowledge, there are no studies correlating the presence of BAT with glucose metabolism parameters in this clinical setting.

### Liver

In order to serve ‘’fight or flight” response, catecholamines are expected to increase glucose output from liver, so that glucose supply to skeletal muscle is amplified. The release of glycolysis-derived lactate and alanine from skeletal muscle (from Cori and alanine cycle respectively), as well as from lipolysis-derived FFAs provides liver with substrates and is well-orchestrated with enzymatic up-regulation of gluconeogenesis and glycogenolysis.

The hyperglycemic effects of catecholamines have been recognized from early hepatic vein catheterization studies in humans [39, 53]. During glucose infusion, adrenaline inhibits splanchnic glucose uptake and prevents the suppression of gluconeogenesis [53]. In addition, at basal state, adrenaline and noradrenaline increase glucogenolysis [39]. The above effects are mediated both directly, by stimulation of hepatic ARs and indirectly, by stimulation of glucagon secretion from the pancreas. Human liver tissue expresses α_1_-AR (specifically α_1Α_ subtype) and β_2_-AR at equal proportions [54, 55]. Both receptors are activated by adrenaline and noradrenaline, however noradrenaline has a higher affinity for the former and adrenaline for the latter [26].

To characterize the direct effects of AR stimulation, the conditions of insulin and glucagon need to be controlled and this can be achieved by somatostatin administration. Under these circumstances, two human studies establish that direct stimulation of α_1_- and β_2_-ARs enhances EGP. The first study, during which adrenaline is administrated at pharmacological doses, reveals a sustained-over time β-AR stimulatory effect in EGP in the absence of α-AR effect [28]. In contrast, the second study demonstrates that, at physiological catecholamine levels, the rise in EGP in post-absorptive phase is mediated by α (and not β)- AR stimulation due to sympathetic neural noradrenaline release [56]. Ex vivo data from transgenic mice show that upon β_2_-AR stimulation, there is increased expression of phosphoenolpyruvate carboxykinase (PEPCK) and glucose-6-phosphatase (G6P). Interestingly, the same study shows that β_2_-AR stimulation is accompanied by glycogen levels depletion, suggesting that glucogenolysis could be, in part, a direct adrenergic effect [57].

Regarding their indirect effects, both catecholamines, especially adrenaline, are well-known stimuli for glucagon secretion [5, 23, 58]. This effect is mediated via activation of both α_1_- and β_2_- ARs in alpha pancreatic cells and involves increase of intracellular calcium (Ca^2+^)_i_ and subsequent exocytocis of glucagon granules [59]. Upon β_2_- AR stimulation by adrenaline, cAMP signaling activates protein kinase A (PKA) and exchange protein directly activated by cAMP (EPAC2), which, in turn, activate two-pore channel 2 (Tpcn2) and liberate nicotinic acid adenine dinucleotide phosphate (NAADP) respectively. This results into Ca^2+^ release from intracellular lysosomal stores, which then triggers further Ca^2+^ release from endoplasmic reticulum (ER) [60]. The exact mechanism by which α_1_-AR stimulation increases (Ca^2+^)_i_ is not clear; it could involve α_1_-AR-induced activation of phospholipase C (PLC), crosstalk with β_2_-AR, or both [61].

However, given that both aforementioned ARs are desensitized after prolonged exposure to catecholamines [36, 37], the above effects might wean off over the course of the disease.

## Insulin secretion

Impaired insulin secretion is the principal cause of hyperglycemia in secretory PPGLs and has been recognized from early case series of patients with pheochromocytomas [38, 58]. The defect of insulin secretion, rather than a matter of cumulative quantity, appears to be a matter of pattern. In particular, there is selective loss of the first phase of glucose-stimulated insulin secretion (GSIS), reflected at insulinogenic index (IGI), accompanied by a delayed rise (breakthrough) of insulin level [62–65].

The inhibitory effect of catecholamines on insulin secretion is mediated via α_2_-AR, for which adrenaline has a greater affinity than noradrenaline [26, 66]. In particular, the α_2A_-AR, the most abundant subtype in pancreatic islets, is key to the process [67]. In response to α_2_-AR agonists, mice exhibit hypoinsulinemia and glucose intolerance. Both effects are exacerbated in transgenic mice with α_2A_-AR overexpression and abolished or eliminated in transgenic α_2A_-AR KO mice or upon α_2_ -AR antagonist administration [68, 69].

While the α_2_-AR-induced inhibition of GSIS is well-established, the impact in basal insulin secretion is less clear, with rodent data ranging from no effect to tonic suppression. Insulin content is no different in pancreatic islets of transgenic mice with either α_2A_-AR overexpression or α_2A_-AR KO, indicating that insulin synthesis is not affected [68, 69].

The underlying mechanism of α_2A_-AR-mediated suppression of GSIS is complex. From early studies in vitro, α_2_-agonists (epinephrine, clonidine), have been demonstrated to decrease (Ca^2+^)_i_ concentration, via either α_2_ -AR agonist-induced membrane repolarisation or inhibition of voltage-dependent calcium channels (VDCCs) though pertussis toxin-sensitive G-protein [70, 71]. However, increasing (Ca^2+^)_i_ level restores insulin release at a minor extent, suggesting that decreased Ca^2+^ influx is not the major operating mechanism [70]. Recent data elucidate another possible mechanism involving nonselective cation channels (NSCCs), which facilitate membrane depolarization by opening background inward currents after glucose-induced closure of the adenosine triphosphate (ATP)-sensitive K^+^ channels (K_ATP_). Adrenaline is demonstrated to inhibit the glucose-induced and incretin-potentiated cAMP production. In addition, it inhibits the activity of transient receptor potential melastatin 2 (TRPM2) channel, a type of NSCCs, in cAMP-dependent manner. In turn, this leads to prolonged lag time and decreased beta cell membrane excitability, and therefore to attenuated insulin secretion. Adrenaline inhibits TRPM2 current exclusively by α_2Α_-AR in the presence of tolbutamide, a sulfonylurea keeping K_ATP_ closed, indicating that the induced effect occurs downstream K_ATP_ [72].

Beyond the clinical setting of secretory PPGLs, α_2_-AR activity upregulation seems to participate in pathogenesis of ‘’wild-type” glucose intolerance. Naturally-occurring single-nucleotide polymorphisms of α_2_ -AR overexpression decrease insulin secretion (both first phase GSIS and basal) and increase the risk of type 2 diabetes mellitus (T2DM) in human and rat, via impaired granule docking at the plasma membrane and beta cell exocytosis, at stage distal to elevation of (Ca^2+^)_i_ [73]. As a general rule, in humans, α_2_ -AR agonists inhibit and α_2_ -AR antagonists enhance insulin secretion [67], with the latter having been studied as potential anti-diabetic agents [74, 75]. However, their net effect in glucose metabolism is blurred by the sympatholytic effect of the former and the sympathogenic effect of the latter [67].

Contrary to the inhibitory effect of α_2_-AR activation, β-AR activation stimulates insulin secretion via inducing adenylate cyclase (AC) activity, increases insulin content and reduces beta cell apoptosis [76, 77].

Apart from their direct effects in pancreatic beta cells, catecholamines affect glucagon-like peptide 1 (GLP-1) expression via their ARs in distal gut. However, their effect depends on AR type, being inhibitory upon α_2Α_-AR stimulation and stimulatory upon α_1_-AR and β_1_-AR stimulation [78, 79]. Considering adrenaline’s preferential affinity for α_2Α_-AR and noradrenaline’s preferential affinity for α_1_-AR and β_1_-AR, a dimorphism of effect in terms of GLP-1 and insulin secretion between adrenaline- and noradrenaline-secreting PPGLs is plausible. In line with this notion, a recent prospective study in patients with pheochromocytomas with adrenergic phenotype, demonstrates impaired first phase of insulin secretion and GLP-1 secretion [65].

Similar to insulin sensitivity outcomes, surgical removal of PPGLs restores insulin secretion more effectively than α-blockade [62, 63], suggesting the existence of further, non-AR-mediated, insulinostatic mechanisms. In fact, apart from catecholamines, secretory PPGLs may co-secrete ectopic hormones and peptides, such as SS, which inhibit insulin secretion [17]. Interestingly, recently published data reveal that 95% of pheochromocytomas express insulin transcript and the hybrid insulin-IGF-2 transcript and that 80% stain positive with anti-insulin antibodies, suggesting that the transcripts are translated to polypeptides [80]. These insulin-related molecules, partly mimicking insulin, may act in bidirectionally mode. They can either block the insulin receptor (IR) leading to hyperglycemia, or activate it, leading to hypoglycaemic attacks, which, are rare yet observed in this clinical setting [81].

## Presentation, prognosis and management of secondary diabetes mellitus in Pheochromocytomas and Paragangliomas (PPGLs)

Being consistent with its pathogenesis, secondary DM in PPGLs is more strongly associated with parameters of impaired insulin secretion than of increased insulin resistance [10]. Furthermore, according to retrospective data, its presentation is also affected by other factors, either patient-specific, such as older age [7, 11, 82], or disease-specific such as longer duration of the disease [11], higher number of anti-hypertensive medications [82], higher levels of metanephrine and normetanephrine [7], larger size of the tumor [8, 9], and presence of PPGL-associated symptoms [9, 11]. In most cases, DM is revealed during routine blood investigations in patients with PPGLs, and sometimes, DM diagnosis precedes that of PPGLs. In a minority of cases, secondary DM may present with acute presentations of DKA [12–14], HSS [15] or hyperglycemia with lactic acidosis [40]. Co-existence of hyperglycemia with hypertension should raise the clinical suspicion of PPGL, especially in young (<50 years) and normal-weight patients [12–14, 40, 83], with negative type 1 diabetes mellitus (T1DM)-related autoantibodies [84].

Few studies have addressed the management of secondary DM in PPGLs. According to a retrospective study of 204 patients with PPGLs and secondary DM, 40% of patients are controlled on diet, 27% of patients receive metformin as monotherapy or in combination with other oral anti-diabetic medications (sulfonylureas or dipeptidyl peptidase 4 inhibitors) and 33% of patients are insulin-treated [7]. Apart from targeting insulin resistance, metformin may have a disease-modifying role in PPGLs, on the basis of anti-proliferative effects shown in vitro, mainly employing the AMPK-mediated inhibition of mTOR pathway [85]; Regarding the newer anti-diabetic medications, GLP-1 receptor agonists (GLP-1RAs) are postulated to be effective in the light of underlying impairment of incretin effect in patients with adrenergic phenotype [65]; albeit, their use should be avoided in patients bearing rearranged-during-transfection (RET) protooncogene mutations, due to risk of medullary thyroid carcinoma [86]. Both GLP-1RAs and sodium-glucose cotransporter 2 inhibitors (SGLT2is) might confer cardioprotective effects in this clinical setting [87]. Intriguingly, the latter are also shown to effectively inhibit SGLT activity and I_K(M)_ electrical current in pheochromocytoma cell lines; the subsequent perturbance of membrane excitability in these cells might have functional implications [88]. However, SGLT2is should be used with caution in insulin-deficient patients due to risk of euglycemic DKA [82]. Similar disease-modifying or pleiotropic effects of anti-diabetic medications have been also demonstrated in other endocrinopathies, as in acromegaly [89] and primary aldosteronism [90].

There are no studies assessing the glycemic efficacy of alpha- and beta-adrenergic blockade in patients with secondary DM due to PPGLs. In few studies investigating its impact in non-diabetic patients with PPGLs, both insulin sensitivity [21] and insulin secretion [62, 63] are improved. Moreover, the preoperative use of metyrosine, an inhibitor of catecholamines synthesis, may decrease insulin requirements by 50% preoperatively [84].

Post-operatively, DM is shown to markedly improve, with complete resolution being observed at 57–79% of cases in cohort studies [7–11] and up to 100% in case reports [16, 40, 84]. Shorter duration of DM [10] or PPGL [82], preoperative DM management with diet and metformin monotherapy [7, 82], and, notably, higher preoperative catecholamine level as well as larger tumor size appear to predict remission of DM [7]. The impact of body weight in post-operative DM prognosis appears to be controversial among studies. While elevated BMI appears to predict DM persistence post-operatively in four retrospective studies [7–9, 11], a third study fails to reveal any correlation between post-operative weight gain and glycated hemoglobulin (HbA1c) [91]. The high rates of DM remission, in conjunction with the risk of post-operative hypoglycemia principally due to excessive rebound hyperinsulinemia require vigorous glucose monitoring post-operatively and appropriate down-titration of anti-diabetic regimens, especially in insulin-treated patients [82, 92].

## Conclusions and future perspectives

Disorders of glucose homeostasis in secretory PPGLs are common, being encountered in almost half of the reported cases; therefore, the co-existence of unexpected, according to age and BMI, hypertension and DM should alert the clinicians to the possibility of such tumors. The aim of this review was to characterize the pathogenesis, presentation, prognosis, and management of secondary DM in this clinical setting.

**Fig. 1:**
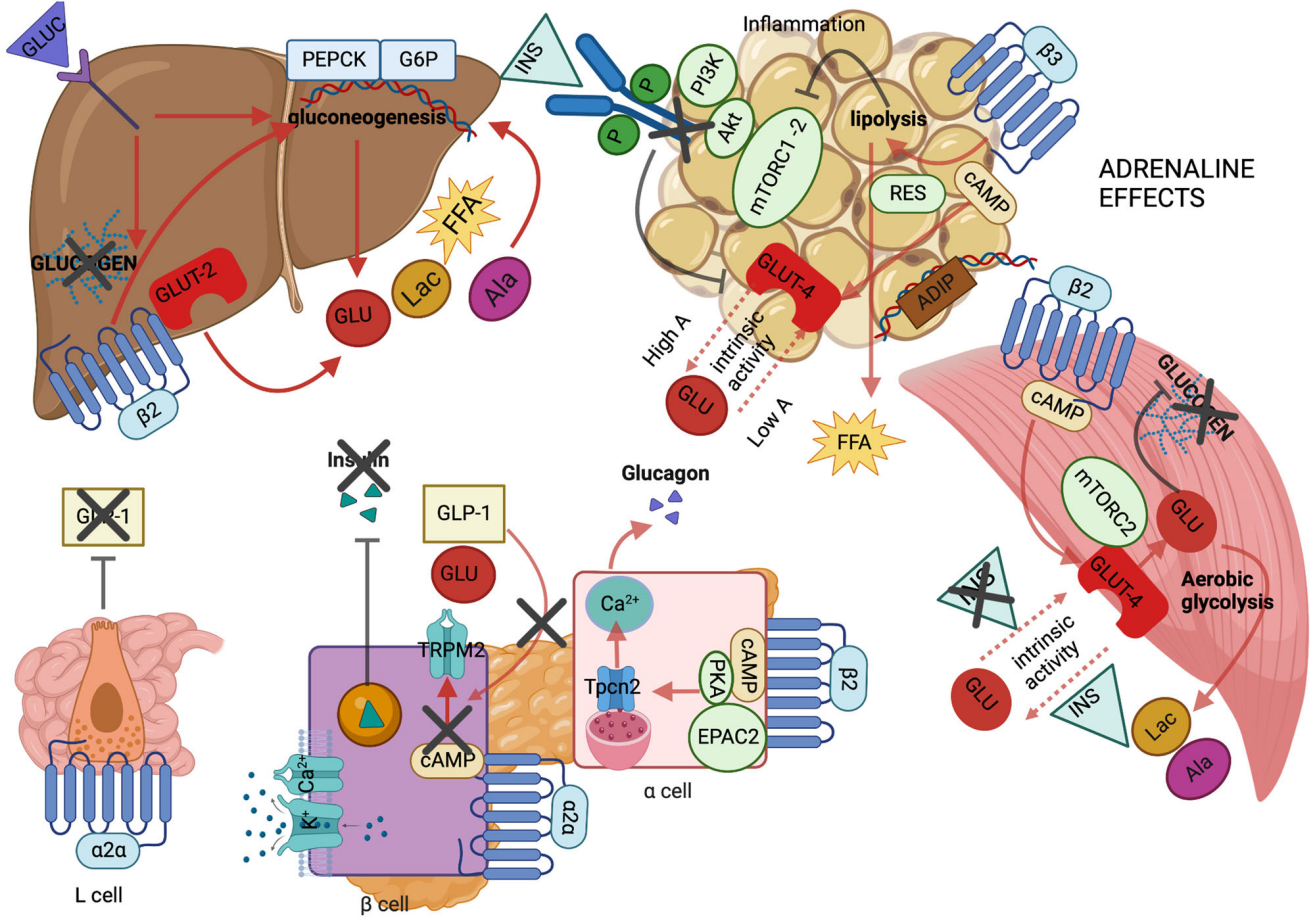
Suggested effects of adrenaline in glucose metabolism. Cross-talk among muscle, adipose tissue, liver, pancreas and gut. In skeletal muscle, beta 2 adrenergic receptor (β2) stimulation increases glucose transporter 4 (GLUT-4) translocation to plasma membrane and glucose (GLU) uptake, via mammalian target of rapamycin complex 2 (mTORC2) and cyclic adenosine monophosphate (cAMP) signaling. The intrinsic activity of GLUT-4 is enhanced in the absence of insulin (INS) and inhibited in the presence of INS. Intracellularly, metabolism is shifted to glycogenolysis and aerobic glycolysis, leading to increased release of lactate (Lac) and alanine (Ala) in the bloodstream. In adipose tissue, beta 3 adrenergic receptor (β3), stimulates lipolysis and free fatty acid (FFA) release in the bloodstream. In turn, lipolytic products induce mTOR complex 1 and 2 (mTORC1-2) dissociation, leading to inhibition of phosphatidylinositol-3 kinase (PI3K) - protein kinase B (Akt) - mTOR pathway and, therefore to decreased insulin-mediated GLUT-4 translocation and GLU uptake. In parallel, β3 affects basal GLU uptake via cAMP signaling, in biphasic mode, depending on adrenaline (A) level. At low A level, the intrinsic activity of GLUT-4 is promoted leading to enhanced GLU uptake, while at high level, GLU uptake is compromised. Meanwhile, downregulation of adiponectin (ADIP), upregulation of resistin (RES) and inflammation further exacerbate insulin resistance. In liver, increased influx of gluconeogenesis substrates, i.e. Lac, Ala and FFA, together with inhibition of glucose uptake by glucose transporter 2 (GLUT-2) and β2-induced expression of phosphoenolpyruvate carboxykinase (PEPCK) and glucose-6-phophatase (G6P) enhance gluconeogenesis, in parallel with increasing glucogenolysis. These effects are further exacerbated by augmented glucagon (GLUC) secretion. The secretion of the latter from alpha pancreatic cells, is upregulated upon β2 stimulation, via cAMP-induced activation of protein kinase A (PKA) and exchange protein directly activated by cAMP (EPAC2), which, in turn, activate two-pore channel 2 (Tpcn2), resulting into increased intracellular calcium (Ca^2+^) and exocytosis of GLUC granules. In beta cells, alpha 2 subtype alpha adrenergic receptor (α2α) induces membrane repolarisation or inhibition of voltage-dependent calcium channels, leading to decreased intracellular Ca^2+^. Additionally, α2α attenuate the glucose-induced and glucagon-like peptide 1(GLP-1) potentiated cAMP production, leading to inhibition of transient receptor potential melastatin 2 (TRPM2) and decreased beta cell membrane excitability. Finally, α2α inhibits GLP-1 production from L-enteroendocrine cells (L-cells). All these phenomena synergistically decrease INS secretion. A-responsive genes are represented by parallelograms, colored light blue if upregulated and brown if downregulated. Intracellular proteins are represented by light green oval shape and cAMP by beige oval shape. Adrenergic receptors are displayed by G-protein coupled receptor transmembrane structure and ion channels are depicted in both closed and open forms, according to the occurring effect. GLU is represented by red oval shape, Lac by ocher oval shape, Ala by fuchsia oval shape, FFA by yellow star-like shape, INS by light blue triangle, GLUC by purple trapezium, and GLP-1 by beige parallelograms. The occurring stimulatory or inhibitory effects are represented by solid arrow (red color) and inhibitor lines (black color); the latter are also demonstrated by black crosses. Dashed arrow lines are used to describe ambiguity of GLUT-4 function, according to its intrinsic activity. A adrenaline, Akt protein kinase B, Ala alanine, ADIP adiponectin, Ca^2+^ calcium/calcium channel, cAMP cyclic adenosine monophosphate, EPAC2 exchange protein directly activated by cAMP, FFA free fatty acid, GLP-1 glucagon-like peptide 1, G6P glucose-6 phosphatase, GLU glucose, GLUC glucagon, GLUT-2 glucose transporter 2, GLUT-4 glucose transporter 4, INS insulin, K^+^ potassium channel, Lac lactate, L cell L enteroendocrine cell, mTORC1-2 mammalian target of rapamycin complex 1-2, mTORC2 mammalian target of rapamycin complex 2, PEPCK phosphoenolpyruvate carboxykinase, PI3K phosphatidylinositol 3-kinase, PKA protein kinase A, TRPM2 transient receptor potential melastatin 2 channel, Tpcn2 two-pore channel 2, α2α alpha 2α adrenergic receptor, α cell alpha pancreatic cell, β2 beta 2 adrenergic receptor, β3 beta 3 adrenergic receptor, β cell beta pancreatic cell

**Fig. 2:**
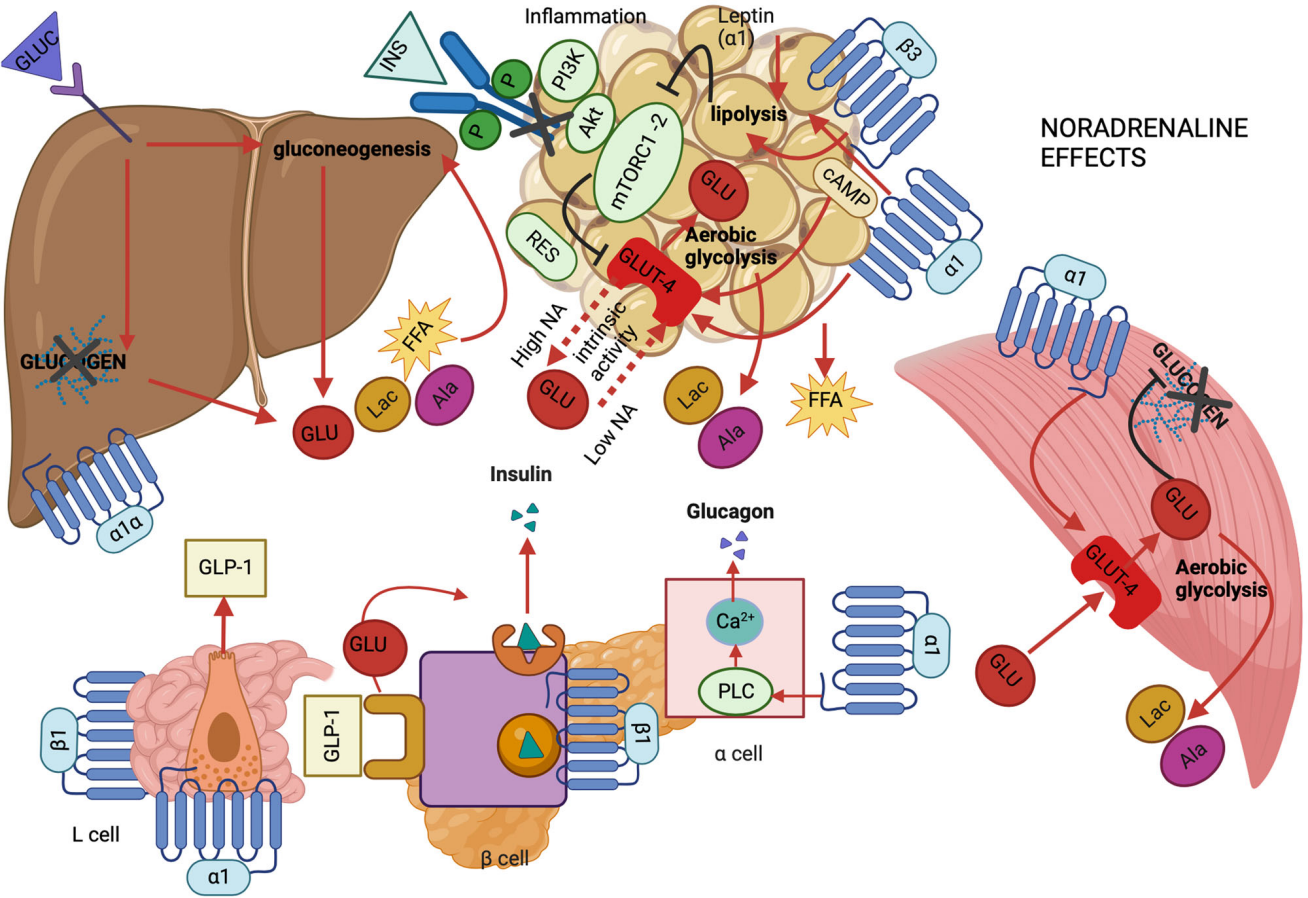
Suggested effects of noradrenaline in glucose metabolism. Cross-talk among muscle, adipose tissue, liver, pancreas and gut. In skeletal muscle, alpha 1 adrenergic receptor (α1) stimulation increases glucose transporter 4 (GLUT-4) translocation to plasma membrane and glucose (GLU) uptake with concomitant glycogenolysis. In adipose tissue, beta 3 adrenergic receptor (β3) and alpha 1 adrenergic receptor (α1) stimulate lipolysis and free fatty acid (FFA) release in the bloodstream. In turn, lipolytic products induce mTOR complex 1 and 2 (mTORC1-2) dissociation, leading to inhibition of phosphatidylinositol-3 kinase (PI3K) - protein kinase B (Akt) - mTOR pathway and, therefore to decreased insulin-mediated GLUT-4 translocation and GLU uptake. In parallel, β3 affects basal GLU uptake via cAMP signaling, in biphasic mode, depending on noradrenaline (NA) level. At low NA level, the intrinsic activity of GLUT-4 is promoted leading to enhanced GLU uptake, while at high NA level, GLU uptake is compromised. By contrast, α1 stimulation enhances GLU uptake. In parallel, α1-induced increased leptin secretion further amplifies lipolysis, while upregulation of resistin (RES) and inflammation further exacerbate insulin resistance. In both muscle and adipose tissue, metabolism is shifted to aerobic glycolysis, leading to increased release of lactate (Lac) and alanine (Ala) in the bloodstream. The increased influx of gluconeogenesis substrates in liver, i.e. Lac, Ala and FFA, together with alpha 1 subtype alpha adrenergic receptor (α1α) stimulation enhance gluconeogenesis and glucogenolysis. These effects are further exacerbated by augmented glucagon (GLUC) secretion. The secretion of the latter from alpha pancreatic cells, is upregulated upon α1 stimulation, possibly via phospholipase C (PLC) activation, resulting into increased intracellular calcium (Ca^2+^) and exocytosis of GLUC granules. In beta cells, we hypothesize that beta 1 adrenergic stimulation (β1) increases insulin (INS) secretion. Finally, in L-enteroendocrine cells (L-cells), α1 and β1 stimulation enhance glucagon-like-peptide 1 (GLP-1) secretion, further augmenting INS secretion. Intracellular proteins are represented by light green oval shape and cAMP by beige oval shape. Adrenergic receptors are displayed by G-protein coupled receptor transmembrane structure. GLU is represented by red oval shape, Lac by ocher oval shape, Ala by fuchsia oval shape, FFA by yellow star-like shape, INS by light blue triangle, GLUC by purple trapezium, and GLP-1 by beige parallelograms. The occurring stimulatory or inhibitory effects are represented by solid arrow (red color) and inhibitor lines (black color); the latter are also demonstrated by black crosses. Dashed arrow lines are used to describe ambiguity of GLUT-4 function, according to its intrinsic activity. Akt protein kinase B, Ala alanine, Ca^2+^ calcium, cAMP cyclic adenosine monophosphate, FFA free fatty acid, GLP-1 glucagon-like peptide 1, GLU glucose, GLUC glucagon, GLUT-4 glucose transporter 4, INS insulin, Lac lactate, L cell L enteroendocrine cell, mTORC1-2 mammalian target of rapamycin complex 1-2, NA noradrenaline, PI3K phosphatidylinositol 3-kinase, PLC phospholipase C, RES resistin, α1α alpha 1α adrenergic receptor, α1 alpha 1 adrenergic receptor, α cell alpha pancreatic cell, β1 beta 1 adrenergic receptor, β3 beta 3 adrenergic receptor, β cell beta pancreatic cell

The key pathogenetic mechanism of secondary DM in secretory PPGLs is impaired insulin secretion, which is more relevant for adrenergic phenotype, due to α_2α_-AR mediated inhibition of insulin and GLP-1 secretion (Fig. 1). Both adrenergic and noradrenergic phenotypes are also characterized by insulin resistance, displayed by impaired glucose utilization, excessive lipolysis, perturbed adipokine expression, inflammation, enhanced gluconeogenesis and glycogenolysis, as well as by stimulation of glucagon secretion (Figs. 1 and 2). A dimorphic DM phenotype based on secretory phenotype is therefore plausible. Patients with noradrenaline-secreting tumors could be managed with diet and metformin or other oral antidiabetic agents, while those with adrenaline-secreting tumors might require close monitoring and timely initiation of either GLP-1RAs or insulin treatment (Table 1). These patients also necessitate prompt insulin down-titration or discontinuation post-operatively. The tendency of adrenergic phenotype towards insulin deficiency explains the preferential use of lipids as energy substrates in comparison to noradrenergic phenotype, which favors carbohydrate utilization. Nevertheless, to the best of our knowledge, there are no studies correlating DM phenotype with PPGLs’ secretory profile, thus this hypothesis warrants further validation in clinical studies, which could also investigate potential correlations with cardiovascular complications. In addition, our pathogenesis understanding derives predominantly from human or animal models of acute catecholamine administration, therefore the impact of desensitization of some types of ARs over chronic exposure cannot be appreciated. Moreover, the efficacy and safety of newer anti-diabetic medications such as GLP-1RAs and SGLT2is necessitates assessment in prospective, cohort studies.

**Table 1:** Hyperglycemia in secretory pheochromocytomas and paragangliomas

Catecholamines	Pathophysiologic mechanisms					
Adrenaline	Insulin sensitivity		Insulin, glucagon & incretin secretion		Glycaemic phenotype	Suggested management
	Muscle (β_2_-AR) *^1^	↑ GLU uptake (absence of insulin), glucogenolysis & aerobic glycolysis ↓ glucogenesis & oxidative phosphoryliosis	Alpha cells (β_2_-AR)*^1^	↑ glucagon	Insulin-deficient DM (rarely: DKA, HSS)	Metformin + GLP-1RAs^*2^ ± Insulin (prompt initiation might be needed)
	Adipose Tissue (β_3_-AR)	↑ lipolysis ↓ GLU uptake & adiponectin expression	Beta cells (α_2α_-AR)	↓ insulin		
	Liver (β_2_-AR) *^1^	↓ GLU uptake ↑ gluconeogenesis & glucogenolysis	L- cells (α_2α_-AR)	↓ GLP-1		
Noradrenaline	Insulin sensitivity		Insulin, glucagon & incretin secretion		Glycaemic phenotype	Suggested management
	Muscle (α_1_-AR) *^1^	↑ GLU uptake^*1^	Alpha cells (α_1_-AR) *^1^	↑ glucagon	IGT, Insulin-resistant DM	Diet + Metformin ± DDP4i/SGLT2i
	Adipose Tissue (β_3_-AR & α_1_-AR) *^1^	↓ or ↑ GLU uptake, ↑glycolysis, lipolysis, leptin & adiponectin secretion	Beta cells	?↑ insulin		
	Liver (α_1α_-AR) *^1^	↑ gluconeogenesis & glucogenolysis	L-cells (β_1_-AR & α_1_-AR)	↑ GLP-1 secretion		

The table is divided in 2 main subsections (raws), corresponding to the 2 main catecholamines secreted by these tumors, adrenaline and noradrenaline. For each of the two catecholamines, the corresponding pathophysiologic mechanisms, glycemic phenotype and suggested management are presented (columns). The pathophysiologic mechanisms of insulin resistance are subclassified as per the 3 insulin-sensitive tissues, namely muscle, adipose tissue and liver, and the effects in insulin, glucagon and glucagon-like peptide 1 (GLP-1) secretion are also subclassified accordingly. The suggested management matches each clinical phenotype and the principal secretory profile. The presence of cardiovascular disease, heart failure and chronic kidney disease has not been considered in suggested management here, but, if present, the current guidelines for type 2 diabetes mellitus (T2DM) with glucagon-like peptide 1 receptor agonists (GLP-1RA) or sodium glucose co-transporter 2 inhibitors (SGLT2i) should be followed.*^1^: these adrenergic receptors are desensitized after chronic exposure, *^2^: only in cases without rearranged-during-transfection (RET) protooncogene mutations ↑: increase, ↓: decrease, +: plus, ± plus or minus *DKA* diabetic ketoacidosis, *DM* diabetes mellitus, *DPP4i* dipeptidyl peptidase 4 inhibitors, *GLU* glucose, *GLP-1* glucagon-like peptide 1, *GLP-1RA* glucagon-like peptide 1 receptor agonist, *HSS* hyperglycemic hyperosmolar state, *IGT* impaired glucose tolerance, *L-cells* L-enteroendocrine cells, *SGLT2i* sodium glucose cotransporter 2 inhibitor, *α*_*1*_ alpha 1 adrenergic receptor, *α*_*1α*_ alpha 1α adrenergic receptor, *α*_*2α*_ alpha 2α adrenergic receptor, *β*_*3*_ beta 3 adrenergic receptor, *β*_*1*_ beta 1 adrenergic receptor [5, 7–16, 23, 26, 28, 32–39, 41–48, 53–61, 65–72, 76–79, 82]

In the era of emerging, personalized, genetic-driven, cluster-specific management of PPGLs, studies linking DM phenotype to the 3 main molecular clusters, would broaden our understanding on genotype-phenotype correlations, possibly beyond the secretory profile of these tumors. For example, it would be particularly interesting to investigate if levels of lactate and alanine are higher in cluster 1 secretory PPGLs (pseudohypoxia-related) than in tumors of the other 2 clusters, in order to understand if anaerobic glycolysis in these PPGLs is limited to the tumor cells or expanded to encompass insulin-sensitive tissues, such as muscle and adipose tissue. Furthermore, exploring the genetic basis and role of insulin expression by tumor cells and BAT in glucose metabolism could shed light to other pathogenetic aspects of secondary DM in this setting.

Secondary DM in PPGLs remits at a high percentage post-surgery; on the other hand, alpha and beta blockade, as well as metyrosine, improve glucose metabolism at a lesser extent; this possibly reflects either that PPGLs affect glucose metabolism through multiple hormonal co-secretion or that the dosage used for blood pressure control is not adequate for glycemic control. The persistence of DM post-operatively reflects either co-existence of other pathogenetic factors, such as increased age or BMI, or persistence of underlying disease. Regarding the latter, the possible disease-modifying effects of metformin and SGLT2is deserve further investigation in preclinical and clinical studies, as their use could be relevant in cases of persistent DM due to metastatic or recurrent disease.

In conclusion, our perspective towards secondary DM in PPGLs should include the existence of 2 distinct glycemic phenotypes. Their biochemical, genetic and cardiovascular classification, alongside with pathophysiology-targeted management, seems to be the only way towards personalized management of secondary DM in PPGLs.

### supplementary information

Publication License
